# Costs and benefits of Papacarie in pediatric dentistry: a randomized clinical trial

**DOI:** 10.1038/s41598-018-36092-x

**Published:** 2018-12-17

**Authors:** Fernanda Bottega, Sandra Kalil Bussadori, Iara Denise Endruweit Battisti, Eusélia Paveglio Vieira, Tiago Szambelan Pompeo, Eliane Roseli Winkelmann

**Affiliations:** 1 Regional University of the Northwestern of Rio Grande do Sul (UNIJUÍ), Ijuí, RS Brazil; 2 Rehabilitation Sciences and Biophotonics Applied to Heath Science, University of Nove of Julho (UNINOVE), São Paulo, SP Brazil; 3 Federal University of Fronteira Sul (UFFS), Cerro Largo, RS Brazil

## Abstract

Papacarie gel is an agent that eliminates the need for local anesthesia and reduces the need for using a drill. However, there is no information regarding the cost per procedure. Therefore we analyzed the cost, per procedure, of Papacarie gel compared to the traditional method (drilling), and performed a comparison between these methods of carious tissue removal. A randomized clinical trial was performed with 24 children with an average age of 5.9 years old. Of these children, 12 were boys and 12 were girls, which resulted in a total of 46 restorations. Patients were separated into: Papacarie group (caries removal with the chemical-mechanical method - Papacarie gel) and Drill group (caries removal with the traditional method - drilling). Values of the materials used in the procedures, heart rate (before, 5 minutes during, and after dental treatment), and the total consultation duration were recorded. A level of significance of 5% was adopted. Papacarie had a lower cost per procedure ($ 0.91) when compared to the traditional method ($ 1.58). Papacarie provided a cost reduction of 42% compared to the traditional method. Using local anesthesia ($ 2.17), the cost reduction increased to 58%. In the procedure using drill + Papacarie ($ 1.37), the cost reduction was 33%. Heart rate, consultation duration, and number of restorations were not statistically different. Papacarie shows an excellent cost benefit for minimally invasive removal of carious tissue and is a feasible alternative for public health care.

## Introduction

Tooth decay is the most common chronic disease of the oral cavity, being complex and multifactorial. These factors include microbiota, cariogenic diets, susceptible hosts, as well as socioeconomic and environmental factors^1^. Tooth decay can be considered one of the major public health problems in Brazil, reaching over 90% of the population above 35 years old^2^. The Brazilian National Household Survey showed that 78% of Brazilian children under 5 years old had never consulted a dentist. Furthermore, over 50% of Brazilian children have already presented at least one decayed tooth, or have lost a tooth by decay or have a restored tooth at 5 years of age^3^. These problems can compromise their quality of life in functional, emotional, and social areas^4^.

There are different methods of caries removal such as mechanical (drilling) and chemical-mechanical. Chemical-mechanical caries removal is a noninvasive technique which eliminates infected tissue, preserving the healthy tooth structure and preventing irritation of the pulp and discomfort to the patient^1^. Minimally invasive techniques have been increasingly used, especially with children^1^. This technique involves removing the decayed tissue via the application of natural or synthetic agents to dissolve and facilitate the removal of the infected tissue. Papacarie stands out among chemical-mechanical techniques^5^. This agent is a gel containing papain and chloramine used in combination with hand tools for minimally invasive removal of carious tissue. This method eliminates the need of local anesthesia and reduces the need for the use of a drill, reducing the discomfort from noise and destruction caused to dental tissue^6^.

Several studies have investigated the effectiveness of this gel^6–9^ and reported satisfactory results regarding the clinical monitoring, anxiety, comfort and pain, acceptance of patients^10,11^, and cost^12^. There were cytotoxicity tests performed of the substance^5,13^ demonstrating its safety for adults or pediatric patients.

Papacarie gel can be used successfully in patients with special needs, pediatric dentistry, and adults with phobias. Its implementation is an important alternative in public health care because it combines practicality, ease of use, low cost and does not require the use of local anesthesia^14^. Most papers published on the effectiveness of this gel reported clinical or microbiological cases praising its technique and benefits over other methods^6,9,10^. This investigation has not found any study in literature showing specific information about the financial cost of a restorative procedure using Papacarie gel when compared to the conventional method.

Thus, this study aimed to analyze the cost per procedure of Papacarie gel compared to the traditional method (drilling) and also perform a comparison between both methods of caries removal and their benefits.

## Materials and Methods

### Informed consent

This study was approved by the research ethics committee of the Regional University of the Northwestern of Rio Grande do Sul (UNIJUÍ) under the case number 1086085. This study was registered at the Brazilian Clinical Trials Registry under the case number RBR-9GGHTB on January 8^th^ 2017. All methods were performed in accordance with the relevant guidelines and regulations. Parents or legal guardians received detailed information about the study, and they signed an informed consent form, which allowed the children to participate in the research. The Ethics Committee of UNIJUÍ, Ijuí, Brazil, adheres to *Plataforma Brasil* and makes study protocols available online at the time of protocol acceptance. The authors confirm that no changes were made to the initial protocols.

### Design of the study, setting and inclusion criteria

This study is a randomized, descriptive and analytical clinical trial. In this study, a group was submitted to the chemical-mechanical treatment of caries removal using Papacarie gel (Papacarie group) and the other group was submitted to the traditional mechanical treatment (Drill group) for caries removal.

Children of both genders, with average age of six years old, enrolled in the first grade of municipal schools in the city of Ijuí-RS were included in this trial. Those children had carious cavity lesions in primary and permanent molars in their dental examination. After dental evaluation, 44 children who met the inclusion criteria were included in the clinical trial. However, the intervention was performed in 24 children who had carious lesions in primary or permanent molars Class I (involving only the occlusal aspect) and Class II (involving occlusal and proximal aspect) without clinical signs or symptoms of pulpal involvement (spontaneous pain). Children were excluded if they did not attend the dental consultation. The initial dental examination of these children was carried out at municipal schools and the intervention was performed in the dental office of UNIJUÍ, Ijuí, Brazil, between August and December of 2015.

### Randomization and study groups

In this study, we proposed a sample size of 24 patients in each group [power (1-beta) of 80%; significance level (alpha) of 5%; non-inferiority limit, d value of 48%]. Patients were randomized by a closed envelope method generated by the research coordinator in a 1:1 ratio to either the chemical-mechanical treatment of caries removal using Papacarie gel (Papacarie group) or the traditional mechanical treatment of caries removal (Drill group).

The interventions were performed by a single operator (dentist, primary researcher). Before the execution of the restorative technique, clinical procedure data were collected to characterize the sample through a semi-structured interview and the children’s resting heart rate was monitored using a digital oximeter (Solmedica, Brazil). These data were collected with the child sitting for at least 5 minutes. Later, the researcher performed the restorative medical procedures. The total consultation duration as well as the heart rate every 5 minutes during the procedure were recorded for both groups. After the intervention consultation, each child and his/her guardian received oral hygiene care guidance. The heart rate of the child was also recorded after the procedure.

The materials and common use equipment were not considered when evaluating the average cost for the dental restorative procedure of both groups. The amount of dental material used in the intervention was recorded. This amount differs according to the method and the clinical need, and it would change the total cost of the restorative procedure. The price and amount of materials consumed as well as the clinical need were evaluated. The need for local anesthesia, the number of drills and their sterilization, the amount of Papacarie gel used, and the restorative material of choice were recorded.

### Caries removal techniques

In the Papacarie group, the method of chemical-mechanical caries removal with Papacarie gel was used. The guidelines of the manufacturer have been followed which were to apply the gel in the cavity and leave it to act for about 30 to 60 seconds. Then, the removal of carious tissue with a dental spoon began and was carried out without applying pressure or making cuts. After reaching the vitreous aspect of the cavity, which means that the decayed tissue was totally removed, the restoration was carried out with glass ionomer cement (GIC).

In the Drill group, the conventional treatment of mechanical removal of carious tissue was carried out using high-speed drill bits (KG Sorensen - N° 1011/1012/1014). The access to the lesion was made, the complete removal of the carious dentin was carried out according to tactile and visual clinical criteria, and the tooth was restored with GIC. The criterion for the use of local anesthesia was painful symptomatology.

### Evaluation of the restorations performed

Thirty days after the intervention, a second consultation was held with another dentist. This professional assessed the restorations and painful symptomology through means of a question to the patient: “did you feel pain in the restored tooth?” This evaluator was blinded to the interventions as he did not know the method that was used to remove the decayed tissue.

### Outcomes

The primary outcome was the material cost. The secondary outcomes included: 1) heart rate; 2) consultation duration; 3) number of restorations; and 4) success of restoration.

### Statistical analysis

For the data analysis, absolute frequencies were calculated to characterize the study sample and the Chi-square test was used to check the similarity between both the Papacarie and Drill groups pre-intervention. The Chi-square test and the Mann-Whitney U test were used to detect differences in clinical outcomes between both groups. Covariance analysis was used to analyze the heart rate pre and post intervention between both groups. The statistical tests considered a 5% level of significance. The Statistical Package for the Social Sciences software (SPSS Inc., Chicago, IL, EUA, version 23) for Windows software was used for all statistical analyses.

## Results

**Figure 1:**
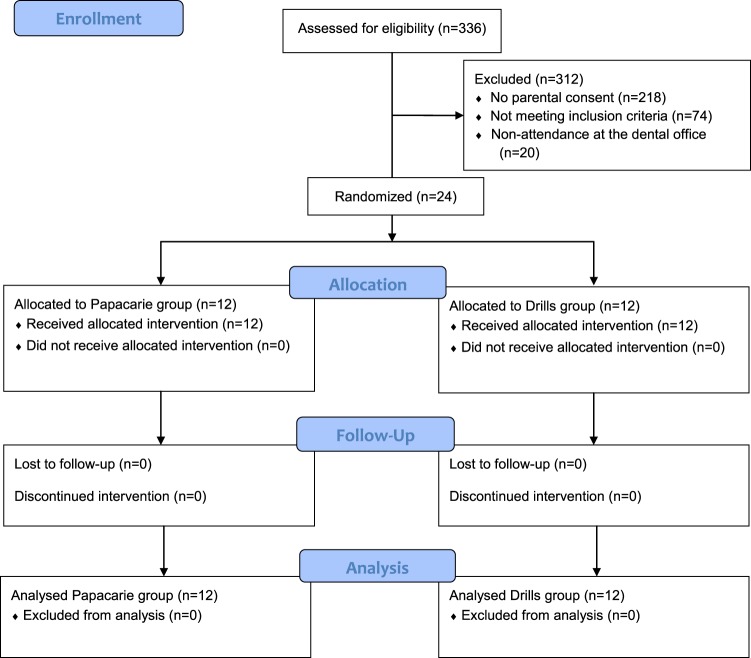
Flowchart of the study design.

Out of a total of 336 students, 118 children were authorized by their parents or responsible guardian to participate in the dental examination. After dental evaluation, 44 children who met the inclusion criteria were included in the clinical trial. However, the intervention was only performed in 24 children who had caries lesions in primary or permanent molars. These children were randomly assigned to the chemical-mechanical treatment of caries removal using Papacarie gel (Papacarie group, n = 12) or to the traditional mechanical treatment (Drill group, n = 12) (Fig. 1). The time from recruitment to follow-up was between August and December of 2015.

Table 1 shows the clinical characteristics and baseline values, which were similar for both groups after randomization. The intervention was performed in 24 children (12 boys and 12 girls), with an average age of 5.9 years old (minimum age of 5 and maximum age of 6).

**Table 1:** Clinical characteristics of the randomized patients.

Characteristics	Papacarie (n = 12)	Drill (n = 12)	p-value
**Gender (male/female)**	5/7	7/5	0.683^&^
**Clinical characteristics of the patients**			
Use of baby bottle (yes/no)	9/3	6/6	0.400^#^
Existence of toothache (yes/no)	9/3	8/4	1.000^#^
Need of endodontics (yes/no)	3/9	2/10	1.000^#^
Need of exodontics (yes/no)	6/6	4/8	0.680^&^
**Dental consultation**			
Have seen a dentist (yes/no)	7/5	10/2	0.371^&^
Service (public/private)	3/4	4/6	–^§^
**Parents’ education (LSE/USE/PSE)**	4/7/1	2/8/2	0.714^&^
**Family income (up to 1 MW/ 1 MW or more)**	8/4	4/8	0.220^&^
**Guardian has knowledge about caries caused by the baby bottle (yes/no)**	0/12	1/11	–^§^

LSE: Lower secondary education; USE: Upper secondary education; PSE: Post-secondary education; MW: Minimum wage; ^&^p-value considering the chi-square test; ^#^p-value considering the Fisher’s exact test; ^§^n < 20, the statistic test was not carried out.

Table 2 describes the clinical results for the Papacarie group and Drill group. There was no statistically significant difference in the clinical outcomes of the intervention. The clinical results, assessed by the blind evaluator, indicate that both restorations had no symptoms of pain and were successful, except for one restoration in the Papacarie group which was evaluated as a failure due to a fracture in the restorative material (glass ionomer cement). Heart rate was increased in both groups, although not significantly different between before and 5 minutes after the beginning of the intervention. This value was maintained until the end of the consultation.

**Table 2:** Clinical results pre and post intervention.

Clinical results	Papacarie (n = 12)	Drill (n = 12)	p-value
**Clinical results of the intervention**			
Duration of the consultation (minimum/median/maximum)	7/12.5/19	7/10.5/18	0.433^#^
Restorations number (Minimum/median/maximum)	0/2/5	1/2/3	0.317^#^
Uncooperative/cooperative patients	8/4	3/9	0.101^&^
**Clinical results of blind assessment** ^§^ **(after 30 days)**			
Successful restoration	25	20	1.000^&^
Failed restoration	1	0	
Excluded tooth	0	0	
Painful symptomatology	0	0	
**Heart rate**			
Before intervention	79.75 ± 7.46	76.08 ± 4.62	0.302*
During intervention (5 minutes after starting)	89.58 ± 12.70	92.25 ± 2.98	
After intervention	86.67 ± 12.26	90.00 ± 5.08	

^#^p-value considering the Mann-Whitney U test; ^&^p-value considering the chi-square test; ^*^p-value considering covariance analysis, pre and post difference between the study groups; ^§^Blind evaluator examined 12 patients of both groups.

Table 3 describes the cost analysis of materials used in each dental restorative procedure for both methods of removing carious tissue. The calculation was based on the evaluation of material costs, excluding commonly used materials in both techniques. The amounts in Brazilian currency (real) were converted into dollars. A reduction of 42% in the cost was seen when using Papacarie gel ($0.91) when compared to the traditional method (drilling). Thus, in a group of 10 patients with these treatment conditions, the traditional method (drilling) would have a total cost of $ 15.85 and using gel would cost $ 9.13. This difference of $ 6.72 could be used for the care of 0.73 more patients. Thus, there would be a 7.30% increase in efficiency. Similarly, the cost of a restoration with Papacarie gel would be 58% cheaper when compared to the cost of a traditional procedure (drilling) using local anesthesia ($ 2.17). In a group of 10 patients, the additional cost for the procedure using drills + anesthesia would be $ 12.63. This value would allow the service of 1.38 more patients, increasing the efficiency by 13.80%. In a clinical procedure that uses drill + Papacarie gel at a cost of $ 1.37, the generated economy efficiency would be 33%. In this situation, the additional cost for 10 patients would be $ 4.56, which would be the cost to treat 0.5 patients, an increase of 5% efficiency.

**Table 3:** Average cost of materials for the dental restorative procedure for both methods of removing carious tissue.

Dental materials	Restauration cost ($)
**Papacarie (syringe 1 mL)**	0.24
**Drill bit (unit)**	0.31*
**Restorative material (GIC)**	0.67
**Sterilization**	
Drill bit	0.15
Instrumental	0.31
**Anesthesia**	0.59^§^
**Restoration**	
Papacarie^®^	0.91
Drill without anesthesia	1.58
Drill with anesthesia	2.17
Drill + Papacarie^®^	1.37

GIC: Glass ionomer cement. ^*^Unit value/10 restorations; ^§^carpule anesthesia – unit value/1000 restorations.

Table 4 describes the analysis of the cost of dental materials, but it also considers the possible clinical variations of different methods of caries removal. A total of 336 children, with average age of 5.8 years, enrolled in the first grade of municipal schools in the city of Ijuí and using public health care were evaluated. A restorative procedure using Papacarie gel would be performed for each of these 336 children resulting in a total material cost of $ 306.71. Considering this total material cost, if the procedure would have been performed with the use of drill + Papacarie gel for caries removal, only 224 children would have been assisted, representing 67% of the children. Also, if a drill would have been used without anesthesia, only 194 patients would be assisted, representing 58% of the children. On the other hand, if drill with anesthesia would have been used, the restorative procedure would have been possible only in 141 of these children, representing only 42% of them. It is noteworthy that when comparing the number of children treated with the drill + Papacarie method, a restoration using only the Papacarie gel could service over 112 children, representing the savings of 33%. So, comparing the use of both drilling without and with anesthesia, Papacarie gel would enable treating 142 and 195 more children respectively, with a cost savings of 42% and 58%, respectively.

**Table 4:** Cost analysis for different methods of caries removal.

Material cost	Papacarie^®^	Drill + Papacarie^®^	Drill without Anesthesia	Drill with Anesthesia
Cost per patient ($)	0.91	1.37	1.58	2.17
Cost for 336 patients ($)	306.71	460.06	532.43	730.58
Difference for 336 patients ($)	—	153.36	225.72	423.88
Difference of assisted patients (n)	336	224	194	141
Not assisted patients (n)	—	112	142	195
Cost savings (%)	—	33.33	42.39	58.02
Increased efficiency (%)	—	0.5	0.74	1.38

## Discussion

In this study, we showed that the chemical-mechanical method (Papacarie) has a lower cost per procedure compared to the traditional method (drill). Papacarie provided a cost savings of 42% compared to the traditional method. Using local anesthesia, the cost savings increased to 58%. In a similar restorative procedure using drill + Papacarie, the cost savings remained at 33%. Heart rate, consultation duration, and number of restorations were not significantly different between the methods of caries removal. The clinical results, assessed by the blind evaluator, indicate that both restorations were successful, with only one failure in the Papacarie group, and with no pain symptoms.

Dental caries are the most common chronic disease in children, and they are of interest to public health care because of their prevalence and treatment costs^2^. Prevention is an important tool because it avoids unnecessary spending on major clinical complications from the caries process. This problem can be avoided by using chemical-mechanical agents, such as Papacarie gel^15^.

Several studies^6,9,11,16,17^ have investigated the effectiveness of this gel compared with the traditional method (drilling) and have reported satisfactory results in the time required for the procedure, clinical monitoring, pain complaints, patient acceptance, and cytotoxicity, which demonstrates its safe use in pediatric patients. Papacarie has proved to be an effective technique in primary and permanent teeth, with a significant reduction of the need for local anesthesia and drill use^6^.

The anxiety in dental treatment has shown that anesthesia and drilling are highly stressful factors. Thus, a non-traumatic method is crucial to avoid fearful and uncooperative patients in dental procedures^11^. Moreover, the knowledge of parents, family environment, and socioeconomic conditions are associated with the collaboration of patients during the treatment^18^. The increased incidence of caries and poor oral hygiene of children are also being attributed to the misinformation of parents^19^. The method of chemical-mechanical removal of caries was developed to overcome these disadvantages by offering more comfort and reducing the stress of the child, therefore resulting in lower clinical complications and adults with no fear of dental care^14^.

Regarding clinical intervention results, there were no significant differences in consultation duration or values of heart rate. Recent literature^20,21^ has shown that Papacarie is associated with longer procedure time; however, our study shows no significant difference. Despite the literature recognizing significant progress in dental treatment, patients bring with them a high level of anxiety^4^. Fear is a natural reaction, which strongly influences patients’ behavior and their cooperation during the procedures. This is a problem especially in pediatric patients who often become a challenge for the professional to treat^22^. Literature^6,7,10^ shows that chemical-mechanical methods act efficiently and have high patient acceptance. Despite a longer time for removal of caries, chemical-mechanical methods can be considered as a feasible alternative especially in pediatric patients.

One month after the dental intervention, both groups had an appointment with the blind evaluator who analyzed the effectiveness of the treatment. It was found that both groups were successful, even though one restoration in the Papacarie group showed a fracture of the restorative material. A previous study^23^ evaluated the success rate after 12 months of follow-up in a series of 84 cases in which chemo-mechanical caries removal was performed with Papacarie and found a failure rate of 12%. Some research^14,18^ reported that the degree of fractures or marginal leakage were related to the properties and clinical limitations of the restorative material, being extremely sensitive to handling and humidity, regardless of the approach. However, this is still the material of choice for non-traumatic restorations due to its ease of use, good adhesiveness, and gradual release of fluoride.

Heart rate may be one of the signs which most expresses anxiety during dental treatment because of the stress common in this situation which stimulates the sympathetic nervous system and consequently releases adrenaline and increases the heart rate^24^. In this study, it can be observed that during the dental care of children, regardless of the treatment group, there were no significant changes in heart rate. The biggest changes, although not significant, occurred at 5 minutes into the intervention and from a clinical point of view, this behavior did not change until the end of the consultation. Anxiety and fear are symptoms usually present in children during dental treatment. The cardiovascular system actively adapts to stress. Cardiovascular responses result mainly in an increase in contractility, cardiac output, and blood pressure^24^. Projective techniques, questionnaires, and physiological signs have been used to assess anxiety in dentistry^19^.

The material cost of a restorative procedure with a chemical-mechanical method with Papacarie gel had greater cost savings when compared with the traditional method with drills. In clinical situations in which local anesthesia would be necessary, or both methods would be needed (drill + Papacarie) in the same procedure, the cost savings would remained. This result was also observed in other studies^9,10,12,13^, although they did not describe the values or cost calculations because neither was the main objective of their studies. The chemical-mechanical agents prevent unnecessary removal of healthy tooth structure, decrease or eliminate the use of local anesthesia, and are more economical compared to all other methods^8^.

Our study has a few potential limitations. First, the resistance of the guardians in taking the children to the dental consultation resulted in a large number of children excluded from this study due to non-attendance at the pre-scheduled appointments. Second, only the total consultation duration was recorded, and not the carious tissue removal duration; therefore, interferences due to non-collaborative patient’s behavior may have influenced the data related to the consultation duration. Third, the lack of consideration of the human resource cost, because only the cost of materials for the dental restorative procedure for both methods of removing carious tissue was recorded.

## Conclusion

The results of this study demonstrated that Papacarie gel shows an excellent cost benefit for minimally invasive removal of carious tissue in children. This method could be recommended for public health care. since it achieves significant reductions in cost for dental restorative procedures and have the same effectiveness as that observed in the traditional caries removal method. Considering there is a significant portion of the population with limited access to dental services and a high tooth decay rate, the use of non-traumatic restorations with Papacarie gel facilitates pediatric dental care and becomes a viable alternative to reduce costs at public health units. Our results showed a short-term analysis. Further studies are needed to evaluate the cost benefit of long-term procedures. In addition, there is a need for education about the importance of children’s oral health care, since there was little involvement of the parents.

## Data Availability

All relevant data are within the paper and its Supporting Information files.
